# Between-centre differences and treatment effects in randomized controlled trials: A case study in traumatic brain injury

**DOI:** 10.1186/1745-6215-12-201

**Published:** 2011-08-25

**Authors:** Hester F Lingsma, Bob Roozenbeek, Pablo Perel, Ian Roberts, Andrew IR Maas, Ewout W Steyerberg

**Affiliations:** 1 Department of Public Health, Erasmus MC, P.O. Box 2040, 3000 CA Rotterdam, the Netherlands; 2 Department of Neurosurgery, Antwerp University Hospital, Wilrijkstraat 10, 2650 Edegem, Belgium; 3 Epidemiology and Population Health Department, London School of Hygiene & Tropical Medicine, Keppel Street, London WC1E 7HT, UK

## Abstract

**Background:**

In Traumatic Brain Injury (TBI), large between-centre differences in outcome exist and many clinicians believe that such differences influence estimation of the treatment effect in randomized controlled trial (RCTs). The aim of this study was to assess the influence of between-centre differences in outcome on the estimated treatment effect in a large RCT in TBI.

**Methods:**

We used data from the MRC CRASH trial on the efficacy of corticosteroid infusion in patients with TBI. We analyzed the effect of the treatment on 14 day mortality with fixed effect logistic regression. Next we used random effects logistic regression with a random intercept to estimate the treatment effect taking into account between-centre differences in outcome. Between-centre differences in outcome were expressed with a 95% range of odds ratios (OR) for centres compared to the average, based on the variance of the random effects (tau^2^). A random effects logistic regression model with random slopes was used to allow the treatment effect to vary by centre. The variation in treatment effect between the centres was expressed in a 95% range of the estimated treatment ORs.

**Results:**

In 9978 patients from 237 centres, 14-day mortality was 19.5%. Mortality was higher in the treatment group (OR = 1.22, p = 0.00010). Using a random effects model showed large between-centre differences in outcome (95% range of centre effects: 0.27- 3.71), but did not substantially change the estimated treatment effect (OR = 1.24, p = 0.00003). There was limited, although statistically significant, between-centre variation in the treatment effect (OR = 1.22, 95% treatment OR range: 1.17-1.26).

**Conclusion:**

Large between-centre differences in outcome do not necessarily affect the estimated treatment effect in RCTs, in contrast to current beliefs in the clinical area of TBI.

## Background

Traumatic brain injury (TBI) is a major health and socio-economic problem throughout the world. It is the field with one of the greatest unmet needs in medicine and public health [1]. Not only is TBI a major cause of death and disability, incurring great personal suffering to victims and relatives, but it also leads to huge direct and indirect costs to society [2].

Many randomized controlled trials (RCTs) have been performed to investigate the effectiveness of new therapies in TBI, but very few have convincingly demonstrated benefit [3]. Multiple factors may have contributed to this disappointing picture, including RCTs in TBI being too small to detect or refute reliably moderate but clinically important benefits or hazards of treatment [4]. To design trials of sufficient size to detect moderate treatment effects, participation of multiple centres is required.

Considerable between-centre differences in patient outcome have been reported in TBI [5-7]. Recently it was shown that a 3.3-fold difference between centres in the odds of having an unfavourable outcome exist (p < 0.001), which was not explained by random variation or patient characteristics [8].

Many clinicians in the field of TBI believe that such between-centre differences in outcome influence the chances of demonstrating a treatment effect in RCTs [7,9]. The aim of this study is to assess the effect of between-centre differences on estimates of the treatment effect in a large RCT in TBI.

## Methods

### Data

We used the individual patient data of the MRC CRASH trial. The CRASH trial (corticosteroid randomisation after significant head injury) is a large, international, randomised placebo-controlled trial of the effect of early administration of 48 h infusion of corticosteroids (methylprednisolone) on risk of death and disability after head injury. Patients from 239 centres in 48 countries were enrolled between April 1999 and May 2004, when the steering committee stopped recruitment because of a higher 14 day mortality rate in the treatment group [10].

### Analysis

We first assessed whether there were differences in outcome between the centres in the CRASH trial, using a random effect logistic regression model (Appendix 1). In this model the outcome of a patient is only determined by the centre that treats the patient. Since some centres only treat a small number of patients, part of the between-centre differences are caused by random variation. The random effect model estimates the between-centre differences beyond random variation. The between-centre differences are expressed as τ^2^, which is the variance of the random effects.

Part of the differences between centres may be caused by the fact that centres are from a particular country. To separate between-centre differences from between-country differences we extended the random effect model with a country level.

Because part of the between-centre effect may be explained by differences in patient characteristics, we adjusted the between-centre differences in outcome for age, Glasgow Coma Scale (GCS) and pupil reactivity at admission. These are the three main generally accepted prognostic factors in TBI [11,12]. Age and GCS (a scale from 1-15) where treated as continuous variables and pupil reactivity as a binary variable (both pupils reactive versus one or both unreactive). So now the outcome of a patient is determined by patient characteristics and centre.

The differences between centres in outcome were expressed in a 95% range of odds ratios for centres compared to the average [13]. To avoid confusion with the odds ratio of the treatment effect we refer to this range as the 95% centre effect range.

Next we estimated the treatment effect with and without taking the between-centre differences into account. We first analyzed the univariate effect of the treatment on 14 day mortality with usual fixed effect logistic regression. Centre effects were ignored, which is a common approach also in multicentre trials. We considered this as the reference strategy.

We furthermore use a random effect model to estimate the treatment effect. The outcome is also determined by the centre, so the treatment effect is adjusted for between centre-differences. This approach assumes a uniform treatment effect across centres. This means we expect the treatment to have equal effects in each centre. As a second approach we used a random effect logistic regression model with interaction between centre and treatment to asses whether the treatment effect varied between the centres. The variation in estimated treatment effect was expressed in a 95% range of the estimated treatment effect across centres. We compared the estimates of the treatment effect and the p-values in the two approaches with the reference strategy.

The random effect estimates of the individual centres for both outcome and treatment effect were plotted with 95% posterior intervals.

Statistical analysis where performed in R statistical software 2.7.2 using the Design and lme4 libraries (R Foundation for Statistical Computation, Vienna). Random effect models were fitted with Adaptive Gaussian Quadrature with 10 qpoints.

This particular analysis did not need ethical approval.

## Results

### Descriptives

In total 10,008 patients were included in the RCT. We excluded 30 patients with missing 14 day outcome, leaving 9978 patients from 237 centres for the analyses. After 14 days 1,948 (19.5%) of the patients had died, with higher mortality in the treatment group. (Table 1)

**Table 1:** Baseline characteristics and 14 day mortality of patients enrolled in the CRASH trial with mortality data available (n = 9978)

	Corticosteroid (n = 4991)	Placebo (n = 4987)
**Age **(median, interquartile range)	33, 23-47	32, 23-47
**Gender**		
Male	4060 (81.3%)	4016 (80.5%)
**Glasgow Coma Scale**		
Severe (3-8)	1966 (39.4%)	1966 (39.4%)
Moderate (9-12)	1554 (31.1%)	1479 (29.7%)
Mild (13-14)	1471 (29.5%)	1542 (30.9%)
**Pupillary reactivity**		
Both reactive to light	4272 (85.6%)	4016 (80.5%)
**14 day mortality**		
Dead	1053 (21.1%)	895 (17.9%)

### Between-centre differences

**Figure 1:**
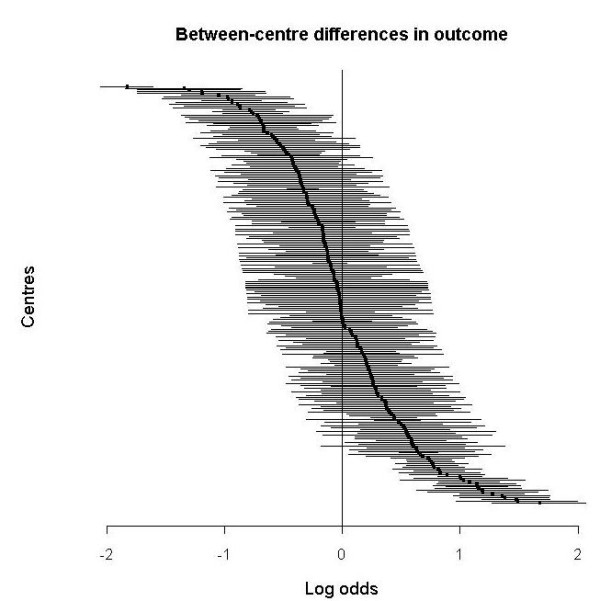
**Differences between centres in mortality, adjusted for age, GCS, pupil reactivity and treatment in a random effects model**. A centre with average mortality has log odds 0, a positive log odds indicates higher mortality. Lines indicate 95% posterior interval.

There was a large difference between centres in outcome (τ^2^_outcome, centre _= 0.447, p < 0.00001). The corresponding 95% range of centre effects was 0.27- 3.71 (Table 2). This means that in centres with the lowest mortality (2.5^th ^percentile) the odds of dying was 0.27 times the average, while in the centres the highest mortality (97.5^th ^percentile) the odds of dying was 3.71 times the average. After adjustment for age, GCS and pupil reactivity the between-centre in outcome increased to τ^2^_outcome, centre _= 0.620 (p < 0.00001) with a corresponding 95% range of centre effects of 0.21- 4.68. Figure 1 shows the estimated adjusted odds ratios for mortality for each centre, compared to the average, with 95% posterior intervals.

**Table 2:** Between-centre and between-country variation in 14 day mortality, unadjusted and adjusted for treatment, age, GCS, and pupillary reactivity

	Unadjusted		Adjusted (Conditional)	
	**Tau**^**2**^	**95% range**	**Tau**^**2**^	**95% range**

**Between-centres**	0.447 (p < 0.00001)	0.27-3.71	0.620 (p < 0.00001)	0.21-4.68
**Between-counties**	0.385 (p < 0.00001)	0.30-3.37	0.642 (p < 0.00001)	0.21-4.81
***Combined: *Between-centres**	0.331 (p < 0.00001)	0.32-3.09	0.235 (p < 0.00001)	0.39-2.58
**Between-counties**	0.142 (p < 0.00001)	0.48-2.09	0.470 (p < 0.00001)	0.26-3.88

Part of the differences in outcome between centres were actually differences between countries. When taking into account that centres are from a particular country, the range of between-centre differences decreased to 0.39-2.58 (τ^2^_outcome, centre | country _= 0.235, p < 0.00001). The range of between-country differences was 0.26 to 3.88 (τ^2^_outcome, centre | country _= 0.470, p < 0.00001).

### Treatment effect

In the reference strategy, the univariate fixed effect logistic regression odds ratio (OR) for treatment was 1.22 (p = 0.0001, Table 3).

**Table 3:** Estimated unadjusted treatment effects (odds ratio (OR) and p value) on 14 day mortality with different approaches taking into account between-centre differences

Approach	Model	OR unadjusted	P value tx effect
-Uniform treatment effect over centres -No adjustment for between-centre differences	Fixed effect logistic regression	1.22	0.00010
-Uniform treatment effect over centres -Adjustment for between-centre differences	Random effect logistic regression with random intercept	1.24	0.00003
-Varying treatment effect over centres -Adjustment for between-centre differences	Random effect logistic regression with random slope	1.22 (95% range: 1.17-1.26)	0.00029

**Figure 2:**
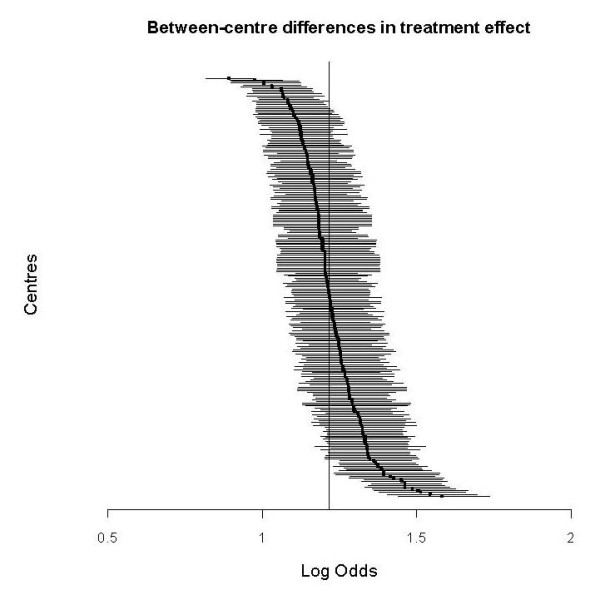
**Between-centre differences in treatment effect, in a random effect model**. The overall treatment effect is log odds = 0.20 (OR = 1.22). Lines indicate 95% posterior interval.

Our first approach of adjusting for the between-centre heterogeneity resulted in an OR for the treatment effect of 1.24 (p = 0.00003). With our second approach we estimated a varying treatment effect between the centres. The mean OR was 1.22 (p = 0.00029). The treatment effect heterogeneity was small, but statistically significant (τ^2^_treaetment effect _= 0.02, p < 0.00001). The corresponding 95% range of the estimated treatment effects across centres was 1.17-1.26 (Figure 2)

## Discussion

Although we found large between-centre differences in outcome in the CRASH trial, taking these into account did not substantially change the estimated treatment effect. Neither did we see major differences in treatment effect by centre. This study provides no support for the hypothesis that between-centre differences in outcome affect the chances of demonstrating a treatment effect in RCTs, in contrast to current beliefs in this clinical area [7,9].

Considering differences between centres in outcome and in estimated treatment effect could be of importance from two perspectives. First, between-centre heterogeneity in the treatment effect between may indicate limited generalizability, which is of importance for example when registering a drug in a particular country. In our study there was no clinically meaningful heterogeneity in overall treatment effect. Although the between-centre differences in the treatment effect were statistically significant, the 95% range was small (1.17-1.26). Clearly, determining generalizability is not solely a statistical issue but requires a clinical judgement to the extent to which the trial results might apply to another population.

Some trials have estimated the heterogeneity of the treatment effect between centres or countries or regions, but did not use random effect modelling. The PLATO study (The Study of Platelet Inhibition and Patient Outcomes) compared two platelet inhibitors (Ticagrelor versus Clopidogrel) for prevention of cardiovascular events in patients with acute coronary syndrome. The overall treatment effect was a hazard ratio (HR) of 0.84 in favour of Ticagrelor. The treatment effect was also tested in four different geographic regions separately; Asia-Australia (N = 1,714), Central-South America (N = 1,237), Europe-Middle East-Africa (N = 13,859), and North America (N = 1,814). In Europe the estimated HR was 0.80 (95% CI: 0.72-0.90). The HRs in Asia-Australia, Central-South America were 0.80 and 0.86, both non statistically significant. The estimated HR in North America was however 1.25 (95% CI: 0.93-1.67). The authors state that "the difference in results between patients enrolled in North America and those enrolled elsewhere raises the questions of whether geographic differences between populations of patients or practice patterns influenced the effects of the randomized treatments, although no apparent explanations have been found."

This interpretation shows the importance to distinct statistical from clinical reasoning. Although the statistical analysis showed significant differences between geographic regions in the PLATO trial, which could be an indication of limited generalizability, the authors have no biological or mechanistic explanation for the heterogeneity of the treatment effect and no heterogeneity was expected on beforehand. In such a situation were region specific estimates of the treatment effect are desired, or when heterogeneity in the treatment effect is expected, we would recommend to use a random effect model to estimate the between-region differences in treatment effect. On the other hand, a limited number of centres, countries or regions, complicates estimation of the heterogeneity in treatment effect.

Second, it is thought that heterogeneity between centres might reduce statistical power to detect the treatment effect.^9 ^Providing that a trial is large enough, randomization will ensure that the intervention and control group are similar with regard to known and unknown confounders [10]. As expected, our study showed that taking into account between-centre differences did not affect statistical significance.

Several explanations can be given for our findings. First, differences in outcome between centres in RCTs may be caused by patient characteristics, which we adjusted for in this analysis. We may not expect that patient characteristics result in differences in treatment effect between centres if the treatment is assumed to work for all patients included in the trial. Secondly there may be differences in care. If these only affect the baseline event rate (e.g. fewer ICU capacity) the treatment effect is not likely to be influenced. In contrast there could be differences in care interacting with the treatment, e.g. if time to hospital arrival is structurally longer in some places, an acute treatment may be less effective. If such an interaction is expected, it would usually be captured in inclusion criteria, such as inclusion within a certain time after injury. In our study we found large differences in outcome between the centres but limited variability in the treatment effect. In other words, there was no substantial interaction between centre and treatment, although such an interaction might have been expected since the CRASH trial comprised an acute treatment and was conducted in low- to high- income countries. This is also an important finding from the perspective of standardisation of care in trials, which some consider very important [9]. Our study suggests that if non-standardized care only influences the absolute risk and does not interact with the treatment, there is no reason to put much effort in standardizing care.

We consider our results to be applicable to drug interventions, which work on physiological mechanisms. Trials investigating a more complex intervention such as surgery or a complex treatment strategy may be more sensitive to differences in quality of care. The effect of outcome difference on treatment effect is not expected to be related to the magnitude of the treatment effect. We recognize that further studies are required to confirm or refute these findings for other types of interventions and for other diseases. Moreover it is crucial to think in advance on the mechanism of the treatment, and whether heterogeneity or homogeneity of the treatment effect by centre is expected.

In this study we have assessed heterogeneity of the treatment effects on a relative scale, but we can also use an absolute scale (risk difference). We found that there is no heterogeneity on the relative scale, despite heterogeneity in the absolute risks per centre. This combination implies that there is heterogeneity in treatment effects on an absolute scale, which is important to realize when considering treatment for individuals [14].

The demonstration of hetero- or homogeneity in treatment effects by country or centre in the single study is conceptually the same as demonstration hetero- or homogeneity is a meta-analysis. The CRASH trial could be seen as a prospective meta-analysis of 40 trials in 40 different countries. A simple way showing the heterogeneity in treatment effects would be to present the results of a forest plot meta-analysis and test for heterogeneity. This was done for the CRASH trial (data not shown), also not indicating heterogeneity.

Our finding that between-centre differences were not explained by patient characteristics corresponds to previous studies in TBI.^8 ^Part of the between-centre differences were actually between-county differences. This could be an indication of centre-differences being caused by structural differences between countries such as availability of resources and organisation of trauma care. The exact explanation of outcome differences between centres and countries requires further study.

Our study has some limitations. First, we did not consider differences in data quality between the centres, which might affect the estimated treatment effect [7]. Second, the CRASH might be considered an exception in the sense that the treatment was harmful. However, it is unlikely that our results would depend on the direction of the treatment effect.

## Conclusion

Our study shows that there were large between centre differences in the CRASH trial, which had no clinically meaningful effect on the estimated treatment effect. Between-centre differences do not necessarily affect the chances of demonstrating a treatment effect, which supports the conduct of large, multi-centre trials.

## Appendix 1

Random effect logistic regression with random intercept for centre

(1) $$\text{Logit }\left(\text{p(}{\text{Y}}_{\text{ij}}=\text{1)}\right)=\beta_{0}+\left({\text{u}}_{0\text{j}}+{\text{e}}_{0\text{ij}}\right)$$

withY_ij _the outcome for patient i in centre j, β_0 _the intercept, u_0j _the random intercept for the centre, and e_0ij _the residuals. The random intercepts are assumed to be normally distributed with τ^2^_0j _= var(u_0j_).

Random effect logistic regression with random intercepts for centre and country

(2) $$\text{Logit }\left(\text{p(}{\text{Y}}_{\text{ij}}=\text{1)}\right)=\beta_{0}+\left({\text{u}}_{0\text{j}}+{\text{u}}_{0\text{k}}+{\text{e}}_{0\text{ijk}}\right)$$

With u_0k _the random intercept for the country, and e_0ijk _the residuals. The random intercepts are assumed to be normally distributed with τ^2^_0j _= var(u_0j_) and τ^2^_0kj _= var(u_0k_).

Random effect logistic regression with random intercept for centre, including patient characteristics

(3) $$\text{Logit }\left(\text{p(}{\text{Y}}_{\text{ij}}=\text{1)}\right)=\beta_{0}+\beta_{\text{1}}{\text{x}}_{\text{ij}}+\left({\text{u}}_{0\text{j}}+{\text{e}}_{0\text{ij}}\right)$$

with patient characteristics x_ij_

Range of the centre effects

(4) $$\text{95}\%\text{ centre effect range}=\text{exp}\left(\text{1}.\text{96}*\tau_{0\text{j}}\right);\text{ exp}\left(\text{1}.\text{96}*-\tau_{0\text{j}}\right)$$

Fixed effect logistic regression

(5) $$\text{Logit }\left(\text{p(}{\text{Y}}_{\text{ij}}=\text{1)}\right)=\beta_{0}+\beta_{\text{1}}{\text{x}}_{\text{ij}}+{\text{e}}_{\text{ij}}$$

with x_ij _the treatment and β_1 _the treatment effect.

Random effect logistic regression with random intercept for centre, including treatment

(6) $$\text{Logit }\left(\text{p(}{\text{Y}}_{\text{ij}}=\text{1)}\right)=\beta_{0}+\beta_{\text{1}}{\text{x}}_{\text{ij}}+\left({\text{u}}_{0\text{j}}+{\text{e}}_{0\text{ij}}\right)$$

with x_ij _the treatment and β_1 _the treatment effect, and random intercept u_0j_

Random effect logistic regression with random slope of the treatment effect per centre

(7) $$\text{Logit }\left(\text{p(}{\text{Y}}_{\text{ij}}=\text{1)}\right)=\beta_{0}+\beta_{\text{1}}{\text{x}}_{\text{ij}}+\left({\text{u}}_{\text{1j}}+{\text{e}}_{\text{1ij}}\right)$$

with u_1j _as the random slope. The random slopes are assumed to be normally distributed with τ^2^_1j _= var(u_1j_)

Random effect logistic regression with random intercept for centre and random slope of the treatment effect per centre

(8) $$\text{Logit }\left(\text{p(}{\text{Y}}_{\text{ij}}=\text{1)}\right)=\beta_{0}+\beta_{\text{1}}{\text{x}}_{\text{ij}}+\left({\text{u}}_{0\text{j}}+{\text{u}}_{\text{1j}}+{\text{e}}_{0\text{ij}}+{\text{e}}_{\text{1ij}}\right)$$

Range of the estimated treatment effect across centres

(9) $$\text{95}\%\text{ treatment effect range}=\text{exp}\left(\beta_{\text{1}}+\text{1}.\text{96}*\tau_{\text{1j}}\right);\text{ exp}\left(\beta_{\text{1}}+\text{1}.\text{96}*-\tau_{\text{1j}}\right)$$

## List of abbreviations

TBI: Traumatic Brain Injury; RCT: Randomized Controlled Trial; OR: Odds Ratio; CRASH: Corticosteroid Randomisation After Significant Head Injury; GCS: Glasgow Coma Scale; HR: Hazard Ratio; ICU: Intensive Care Unit

## Competing interests

The authors declare that they have no competing interests.

## Authors' contributions

HL and BR performed the statistical analyses and drafted the manuscript, with help of ES. PP and IR provided the data and made important intellectual contributions. ES and AM designed the study and made important intellectual contributions. All authors read and approved the final manuscript.
